# Progress in Ophthalmology

**Published:** 1902-05-03

**Authors:** 


### PROGRESS IN OPHTHALMOLOGY.

(*Continued from page* 67.)

**On the Comparative Value of the Preparations of Silver in Ophthalmic Work.**—Gustavus Hartridge[2] at the same meeting read a paper on the above subject, and said:—It is well known that for the treatment of conjunctivitis we possess no specific, and we could hardly expect to have one when we consider the cause, symptoms, and pathological changes that take place in inflammation of a mucous membrane. Hence almost every antiseptic and astringent in the *Pharmacopœia* has been tried at different times. The remedies now most frequently employed are boracic acid, sulphate of zinc, chloride of zinc, sulphate of copper, alum, tannic acid, borax, and nitrate of silver. For many years past the one drug upon which reliance has been placed in all severe cases of purulent ophthalmia is nitrate of silver, and for this purpose it has proved invaluable. The efficiency of the drug depends probably chiefly on its germicidal properties supplemented by its powerful astringent effects upon the vessels of the conjunctiva. The germicidal action is due to the base, silver. The employment of nitrate of silver requires great care and experience, and its use is accompanied by certain disadvantages and limitations, which may be summed-up as follows: Great pain and irritation, strong caustic effect. Long-continued use may produce permanent staining of the conjunctiva. The action of the drug is superficial owing to the ready manner in which it is precipitated by albumen and chlorides. The pain produced by the drug is acute, and may last for some hours even when cocain is used. The pain is generally less and lasts a shorter time when there is a profuse purulent discharge. The silver then becomes quickly converted into an albuminate of silver, a salt which is unirritating. This chemical change in the drug limits its action and prevents it penetrating deeply into the tissue, and thus many organisms escape destruction. The caustic action of the drug may cause sloughing of the epithelial layer of the conjunctiva, and sometimes even of the deeper layers, and so produce permanent cicatrices. On account of these drawbacks efforts have been made to introduce some salt or compound of silver which may be as efficacious as the nitrate, while free from its disadvantages. The following compounds have been supplied to us by the manufacturing chemist: Actol, itrol, argonine, argentamine, nargol, largin, and protargol. He dismisses these one by one, for various reasons, till he comes to protargol, of which he says: It contains 8·3 per cent. of silver, and is not decomposed by albumen, alkalies, or weak hydrochloric acid; very soluble in hot or cold water, but requires protecting from the light, and should be freshly prepared, otherwise it is sticky to use, and becomes very dark coloured. Experiments have proved that protargol is a weaker germicide than nitrate of silver. A 10 per cent. solution of protargol equals in its germicidal effects a 2 per cent. solution of nitrate of silver, but it has a much greater penetrating power and can therefore exert its germicidal qualities longer than the nitrate, the action of which soon becomes stopped by the decomposition which takes place. Protargol is pleasant to use, has no caustic action, and causes little or no pain; it may be applied either as drops or brushed over the mucous membrane by means of a camel-hair brush or a cotton-wool swab. When he first commenced the use of protargol, he began with solutions of 2, 5, and 10 per cent., but soon found that much better effects were produced with 10, 20, and 30 per cent. solutions; now in cases of purulent conjunctivitis he swabs over the conjunctiva twice in 24 hours with 30 per cent. and even where the cornea is implicated this is no contra-indication, the secretion quickly diminishes, the swelling of the lids goes down, and the case rapidly gets well. The good effects appear to be due to the double action of the drug, first its germicidal action, penetrating deeply into the tissue and so exerting its destructive influence on many of the bacilli which would otherwise escape; and secondly to its astringent effect on the conjunctival vessels. In acute contagious conjunctivitis due to the bacillus of Weeks, he found the drug give excellent results. In trachoma he used the drug in very strong solution (50 per cent.), and it soon diminished the secretion and shortened the course of the disease. In chronic conjunctivitis he prefers the zinc salts and mercurial ointment. In suppurative affections of the lachrymal apparatus the drug is especially useful after the canaliculus has been slit up. The sac and nasal duct may be syringed out with a 5 per cent. solution, and if this is well borne the strength should be increased to 10 per cent. Dr. Darier said that having tried all the silver preparations he was convinced that protargol was the most useful; for four years he had used protargol, and during that time he had better results than formerly, so now he never used nitrate of silver at all.

[2] Brit. Med. Jour., Nov. 2, 1901.

